# Linking the degree of intrauterine growth restriction to alterations in metabolism, energy reserves, and gut health in newborn piglets

**DOI:** 10.1093/jas/skag258

**Published:** 2026-08-17

**Authors:** Pau Salgado-López, Júlia Suppi, Katelyn N Gaffield, Mike D Tokach, Jaume Coma, Josep Gasa, David Solà-Oriol

**Affiliations:** Department of Animal and Food Science, Animal Nutrition and Welfare Service (SNIBA), Autonomous University of Barcelona, Bellaterra 08193, Spain; Department of Animal and Food Science, Animal Nutrition and Welfare Service (SNIBA), Autonomous University of Barcelona, Bellaterra 08193, Spain; Department of Animal Sciences and Industry, College of Agriculture, Kansas State University, Manhattan, KS 66506-0201, United States; Department of Animal Sciences and Industry, College of Agriculture, Kansas State University, Manhattan, KS 66506-0201, United States; Vall Companys Group, Lleida 25191, Spain; Department of Animal and Food Science, Animal Nutrition and Welfare Service (SNIBA), Autonomous University of Barcelona, Bellaterra 08193, Spain; Department of Animal and Food Science, Animal Nutrition and Welfare Service (SNIBA), Autonomous University of Barcelona, Bellaterra 08193, Spain

**Keywords:** birth weight, gut development, intrauterine growth restriction, metabolomics, neonatal traits, piglets

## Abstract

In modern swine production, increasing litter size has reduced average birth weight (BtW) and increased the prevalence of intrauterine growth restriction (IUGR). This study aimed to classify piglets by degree of IUGR and evaluate associated morphological, physiological, and behavioral traits. A total of 1,990 piglets were categorized by BtW as IUGR (<0.75 kg), restricted low BtW (R-LBW; 0.75–0.95 kg), non-restricted low BtW (NR-LBW; 0.95–1.15 kg), and normal (N; ≥1.15 kg). A subset of 48 piglets (12/category) was euthanized within 24 h of birth for tissue and blood collection. Data were analyzed by linear mixed models, with piglet category as a fixed effect. Piglets with IUGR had the lowest body weights from birth to weaning, not differing from R-LBW piglets at d 7 and weaning, while N piglets were the heaviest (*P *< 0.01). Average daily gain was lower (*P *≤ 0.03) in IUGR and R-LBW piglets compared with NR-LBW and N piglets throughout lactation. Colostrum intake (CI) differed (*P *< 0.01), with IUGR piglets consuming 285 g less than N piglets. Consequently, NR-LBW and N piglets showed higher immunoglobulin G concentrations compared with the other categories (*P *≤ 0.03). Morphological indicators confirmed growth restriction, with IUGR piglets showing the lowest values. Vitality scores were lowest in IUGR piglets (62.4% scored worst; *P *< 0.01). Piglets with IUGR had lower albumin concentrations than N piglets (*P *< 0.01), whereas N piglets exhibited the highest insulin-like growth factor I concentrations (*P *< 0.01). Metabolomic profiling revealed distinct clustering of IUGR piglets compared with the other categories (*P *≤ 0.01), indicating altered amino acid and energy metabolism. Glycogen concentrations were impaired in IUGR piglets, averaging ∼55% lower than in N piglets (*P *= 0.02). Intestinal function and development were altered in IUGR and low BtW piglets, which exhibited a lower villus height: crypt depth ratio compared with N piglets (*P *≤ 0.03), along with a gene expression profile characterized by upregulation of genes associated with inflammatory response and stress. In conclusion, IUGR piglets exhibited impaired growth during lactation, which was associated with low vitality, reduced CI, metabolic alterations, gastrointestinal dysfunction, and limited energy availability at birth. Further indicators should be investigated to better understand the differences among low BtW piglets, which also exhibited poorer growth performance than N piglets.

## Teaser

Accurate characterization of piglets across different birth weights provides detailed morphological, behavioral, and physiological insights into varying degrees of intrauterine growth restriction, thereby supporting targeted interventions for pigs at risk of growth retardation or poor survivability.

## Introduction

The number of pigs weaned per sow per year is a critical determinant of economic efficiency in swine production. In modern production systems, increasing litter size and the number of piglets weaned per sow are major breeding and management objectives (Hansen et al. 2019), with producers expecting up to 30 pigs weaned per sow annually (Knox 2005). However, it is widely reported that the increase in litter size has resulted in uterine crowding and greater nutrient demands during gestation (Foxcroft et al. 2009). Consequently, average birth weight (**BtW**) has decreased, resulting in a higher proportion of lightweight piglets at birth (Quiniou et al. 2002; Beaulieu et al. 2010; Riddersholm et al. 2021) that are subject to varying degrees of intrauterine growth restriction (**IUGR**; Amdi et al. 2013; Hansen et al. 2019). Wu et al. (2006) defined IUGR as impaired fetal growth during gestation, primarily caused by placental insufficiency, which limits the appropriate supply of oxygen and nutrients to the fetus (Cohen et al. 2015). It is estimated that approximately 20% of piglets within a litter exhibit naturally occurring some degree of IUGR (Krueger et al. 2014).

Intrauterine growth restriction is a key factor that negatively affects overall pig production efficiency (Wu et al. 2006). It is associated with an increased risk of neonatal morbidity and mortality, as well as long-term consequences of fetal programming, including stunted postnatal growth, reduced feed conversion efficiency (Wu et al. 2006; Krueger et al. 2014), and poor carcass quality (Foxcroft et al. 2007). Although fetal and birth weight relative to gestational age are often used to define IUGR (Wu et al. 2006), several studies have emphasized that morphological characteristics provide an additional and necessary criterion for identifying IUGR piglets (Baxter et al. 2008; Douglas et al. 2016; Riddersholm et al. 2021). Reported features include distinctive head morphology (dolphin-like head shape, bulging eyes, and wrinkles perpendicular to the mouth; Chevaux et al. 2010; Hales et al. 2013; Hansen et al. 2019), low body mass index (**BMI**) and ponderal index (**PI**; Hales et al. 2013; Douglas et al. 2016), reduced crown-to-rump length (**CRL**; Salgado-López et al. 2025), and a thin body profile (Baxter et al. 2008; Hales et al. 2013; Riddersholm et al. 2021). While severely IUGR piglets exhibiting all these traits are unable to survive, a major challenge for the pig industry lies in identifying piglets that lack these morphological characteristics at birth but subsequently display poor growth, persistently remaining in the lower quartile of the body weight (**BW**) distribution (Montoro et al. 2020).

In addition to these morphological traits, IUGR piglets prioritize brain development as part of a fetal adaptive response to placental insufficiency (Roza et al. 2008; Amdi et al. 2013), which occurs at the expense of normal development of other organs (Lynegaard et al. 2020). Consequently, IUGR piglets are characterized by gastrointestinal dysfunction (Xu et al. 1994; Yeung and Smyth 2003; Wang et al. 2005), impaired liver development with reduced glycogen concentrations (Vanden Hole et al. 2019), and a lower number of muscle fibers due to a decreased ratio of secondary to primary fibers (Handel and Stickland 1987; Cerisuelo et al. 2007) compared with their normal counterparts. In terms of behavioral variables, Amdi et al. (2013) reported that IUGR piglets consume less colostrum within the first 12 h after birth compared with normal piglets. This reduction has been linked to lower vitality immediately after birth (Herpin et al. 1996; Muns et al. 2013), which compromises the piglet’s ability to effectively ingest colostrum (Oliviero et al. 2019).

Nevertheless, most of these morphological, physiological, and behavioral traits have been well characterized primarily in comparisons between severely IUGR and normal piglets. Developing effective management and nutritional interventions for pigs at risk of growth retardation or poor survivability requires reliable tools to identify piglets affected by any degree of IUGR (Amdi et al. 2013). We hypothesized that an accurate characterization of piglets across different BtW could provide detailed physiological insights and valuable information on piglets experiencing varying degrees of IUGR, thereby enabling the establishment of distinct categories of piglets at birth according to their degree of growth restriction. The present study aimed to develop such a classification, facilitating the implementation of targeted strategies for piglets at higher risk. To achieve this, we comprehensively evaluated morphological, physiological, and behavioral traits across piglets with varying BtW to identify early-life indicators associated with impaired growth and development.

## Materials and methods

The study was approved by the Animal Ethics Committee of the Universitat Autònoma de Barcelona (CEEAH2788M2) and was conducted in accordance with European Union guidelines for the care and use of animals in research (European Parliament 2010).

### Animals, diets, and housing

This experiment was conducted on a 1,050-sow commercial farm (Landrace × Yorkshire) in Spain. A total of 1,990 live-born crossbred piglets (Pietrain × [Landrace × Yorkshire]; 994 females and 996 males) from 102 litters across 11 consecutive batches were included. Sow parity ranged from 1 to 9 (3.5 ± 2.09). The mean litter size was 22.2 ± 4.18 piglets, and the mean BtW was 1.24 ± 0.32 kg.

Sows were fed commercial pelleted diets. During gestation, diets consisted of corn, wheat, and wheat middlings (15.5% crude protein), providing 2,200 kcal of net energy per kg of feed and 0.50% standardized ileal digestible (**SID**) Lys. During lactation, diets were primarily composed of corn, wheat, wheat middlings (15.5% crude protein), and soybean meal, supplying 2,412 kcal of net energy per kg of feed and 0.80% SID Lys. Each female received 2.2 kg of feed per day throughout gestation (from d 30 until placement in the farrowing pens). During the pre-farrowing period, gilts and sows received 2.6 and 3.0 kg of feed per day, respectively. From the day after farrowing, feed allowance increased by 400 g per day until d 9 post-farrowing, after which females were fed ad libitum, reaching a maximum intake of up to 9 kg of feed per sow per day.

The sows were housed in individual farrowing pens (2.7 × 1.7 m^2^), starting 5 d before the expected farrowing date. Farrowing pens had slatted metal flooring in the sow area and slatted plastic flooring in the rest of the pen, with a heating plate provided for the piglets. During the first few days after parturition, a heat lamp was placed above the heating plate. The temperature in the farrowing room was maintained at 24 °C using electronically controlled forced ventilation, with natural light supplemented by artificial lighting from 0600 to 1400 h. During farrowings, the lights remained on until the last monitored sow had completed farrowing, typically around 0100 or 0200 h.

### Experimental design

On the day of birth (d 0), prior to litter equalization, piglets were collected, individually ear-tagged (LeeO®, MSD Animal Health, Barcelona, Spain), and weighed. Piglets were classified based on their BtW as follows: IUGR (BtW < 0.75 kg), restricted low BtW (**R-LBW**; BtW ≥ 0.75 kg and < 0.95 kg), non-restricted low BtW (**NR-LBW**; BtW ≥ 0.95 and <1.15 kg), and normal BtW (**N**; BtW ≥ 1.15 kg). The IUGR category included piglets with a BtW more than 1.5 SD below the mean litter weight (Wang et al. 2016; Van Tichelen et al. 2021), corresponding to a BtW of less than 0.75 kg in the study population. The 1.15 kg cut-off value used to define low BtW was established based on previous literature (He et al. 2016; Wang et al. 2017; Montoro et al. 2020) and the lower 30th percentile of the BtW distribution in the study population. Within the low BtW population without morphological signs of IUGR, two subcategories were defined to reflect different degrees of growth restriction, with the cut-off set as the mean of the lower (0.75 kg) and upper (1.15 kg) thresholds, resulting in a value of 0.95 kg. Piglets with a BtW ≥ 1.15 kg were classified as normal and served as the reference category.

Within 24 h of birth, a subset of 48 piglets (12 per category; one from each category per sow across 12 sows) was euthanized. Half of the sows (*n* = 6) from which piglets were euthanized were of parity 1 to 3 (2.0 ± 0.89), while the other half (*n* = 6) were of parity 4 to 6 (5.3 ± 0.82). Within each category, an equal number of males (*n* = 6) and females (*n* = 6) were selected, except for R-LBW piglets, for which 7 of the 12 euthanized piglets were females, as no additional males were available in this category among the selected sows. To identify severely IUGR piglets exhibiting clear signs of restriction, head morphological characteristics (Chevaux et al. 2010; Hales et al. 2013; Hansen et al. 2019) were evaluated at birth. Severe IUGR piglets were defined by the following morphological characteristics of the head: pronounced, dolphin-like head shape, bulging eyes, hair with no direction of growth, and wrinkles perpendicular to the mouth. Only IUGR piglets presenting all these characteristics were selected for euthanasia, whereas piglets selected from the other three categories showed none of these characteristics.

### Management routines

Cross-fostering was performed within the first 24 h after birth, once it was ensured that all piglets had consumed colostrum. Special care was provided to IUGR piglets within 1 h of birth to stimulate them to start suckling. The cross-fostering protocol for this study included the following steps: 1) all litters were equalized to 15 piglets to ensure each piglet had access to a teat, 2) when litter size exceeded 15 piglets, those with the lowest and highest BtW were fostered to off-test sows, 3) if more than 15 piglets still remained after step two, average-weight piglets were fostered to multiparous sows (parity 2–5), and 4) piglets were only fostered into litters with fewer than 15 piglets on d 1 of life. Steps two and three were designed to maintain approximately half of the litter above the average BtW, stimulating colostrum and milk production by the sow, while ensuring that the remaining half, with below-average BtW, had access to a teat and thus reducing within-litter competition. Overall, this protocol aimed to maximize the number of piglets remaining with their biological sow. Tail docking and iron injection (Uniferon®, Intervet Internation B.V., Boxmeer, The Netherlands) were performed on d 3 after farrowing. Piglets had ad libitum access to water, and creep feed (crude protein = 16.91%, ether extract = 4.8%, lactose = 7.69%, net energy = 2,423 kcal/kg, and SID Lys = 1.2%) was offered ad libitum starting 10 d before weaning. Piglets were weaned 4 wk after farrowing (26.1 ± 0.44 d). Apart from the measurements recorded for this study, all animals were managed according to standard farm routines. The same management routines were applied to all litters across batches to avoid any influence on piglet growth performance and survival.

### Data and sample collection

All piglets were assessed for morphological, behavioral, and physiological traits. At birth, sex, BtW, and CRL were recorded, and rectal temperature (**RT**) was measured within 30 min. The BMI of each piglet was calculated as:


$$\text{BMI}=\left(\text{BtW}\left(\text{kg}\right)/\left[\text{CRL}{\left(m\right)}^{2}\right]\right).$$

The PI of each piglet was calculated as:


$$\text{PI}=\left(\text{BtW}\left(\text{kg}\right)/\left[\text{CRL}{\left(m\right)}^{3}\right]\right).$$

The relative birth weight (**RBW**) of each piglet within a litter was calculated as:


RBW=[(piglet BtW−mean BtW of litter)/mean BtW of litter]×100).

Vitality was scored on a scale from 0 to 3 based on udder stimulation (**U**, score of 0 or 1) and the number of completed circles (**NCC**, score of 0, 1, or 2) around the limits of the bucket (55-cm diameter × 60-cm height, solid plastic enclosure, open at the bottom and top), according to Muns et al. (2013). For U, a score of 0 was assigned when the piglet showed no head movements (e.g., no U or searching behavior within 30 s), and a score of 1 when head movements were observed. For NCC, a score of 0 was assigned when the piglet was unable to rotate its body axis 360° from its initial orientation and could not walk along the limits of the bucket; a score of 1 when the piglet was able to either rotate 360° or walk once along the limits within 30 s; and a score of 2 when the piglet was able to either rotate 360° or walk along the limits at least twice within 30 s. Piglets were weighed again on d 1 (after cross-fostering), d 7, and at weaning. Piglet BW gain within the first 24 h after birth was used to estimate individual colostrum intake (**CI**), based on the equation developed by Theil et al. (2014). Survival was recorded throughout lactation.

The mean ± SD BtW of piglets selected for physiological analyses (*n* = 12 piglets/category) were: 0.57 ± 0.114 kg for IUGR, 0.86 ± 0.067 kg for R-LBW, 1.02 ± 0.051 kg for NR-LBW, and 1.36 ± 0.086 kg for N piglets. First, the piglets were sedated by intramuscular injection of azaperone (Sediron 40 mg/mL; Livisto Int’l, S.L., Barcelona, Spain) and ketamine (Ketamidor 100 mg/mL; VetViva Richter GmbH, Wels, Austria). Subsequently, blood samples (10 mL) were collected from the jugular vein using BD Vacutainer plastic serum tubes (Becton, Dickinson and Company, New Jersey, USA). Samples were kept on ice for 1–2 h, centrifuged at 2,000 × *g* for 10 min at 4 °C to separate serum, and stored at −80 °C until analysis. Because of the limited blood volume collected from IUGR piglets, only six samples were available for serum metabolite analyses in this category. The animals were humanely euthanized by intravenous injection of sodium pentobarbital (Euthasol 400 mg/mL; Dechra Pharmaceuticals PLC, Northwich, United Kingdom), after which tissue samples from the liver, jejunum, and ileum were collected. The liver sample (∼60 g) consisted of pooled pieces from each of the six lobes (lobus sinister lateralis and medialis, lobus dexter lateralis and medialis, lobus quadratus, and lobus caudatus). Immediately after dissection, the samples were snap-frozen and stored at −80 °C until glycogen concentration analysis. Portions of jejunal and ileal tissue (∼1.5 cm^2^) were collected at 30 and 2 cm from the ileo-cecal valve, respectively. Jejunal sections were rinsed in phosphate-buffered saline (**PBS**) and immediately snap-frozen in 1.5 mL of RNAlater (Deltalab, Rubí, Spain), then stored at −80 °C until gene expression analysis. Ileal sections were also rinsed in PBS, placed in individual tubes containing formaldehyde solution for histomorphology analysis, and maintained at room temperature.

### Serum immunoglobulin G, albumin, and insulin-like growth factor I concentrations

Serum samples were analyzed for immunoglobulin G (**IgG**) using Pig IgG ELISA kit (ref. E101-104, Bethyl Laboratories, Inc., Montgomery, Texas, USA), albumin using OSR reagents (Bromoscresol Green Method) with AU480 analyzer (Beckman Coulter®, Krefeld, Germany), and insulin-like growth factor I (**IGF-I**) concentrations using an IGF-I ELISA kit (Ref. E20, Mediagnost, Reutlingen, Germany). Analyses were conducted according to the kit’s manual. ELISA plates were read at 450 nm using the Multiskan Sky and software SkanIt 6.0.1 (ThermoFisher Scientific, Waltham, Massachusetts, USA).

### Serum metabolite analysis

For nuclear magnetic resonance (**NMR**) sample preparation, an aliquot (500 µL) of each serum sample was thawed on ice, vortexed, and centrifuged through 0.5-mL 3 kDa Amicon® ultracentrifuge filters (Merch KgaA, Darmstadt, Germany) at 13,000 × *g* for 60 min at 4 °C. Prior to use, the filters were prewashed four times with 500 µL of HPLC-grade water by centrifugation at 13,000 × *g* for 5 min at 4 °C. The eluted fraction (300 µL) was then mixed with 100 µL of PBS (containing 10% D_2_O with 0.33% DSS, pH 7.4; Merch KgaA, Darmstadt, Germany) and transferred into a 5-mm Wilmad® NMR tube (Merch KgaA, Darmstadt, Germany). An additional 150 µL of D_2_O was added prior to acquisition of the ^1^H NMR profile.

^1^H NMR spectra were obtained using a Bruker 600-MHz Avance III NMR spectrometer (Bruker Biospin, Rheinstetten, Germany) operating at a ^1^H frequency of 600.13 MHz. The sample temperature was set to 298 K, with an equilibration period of 10 min prior to each spectra acquisition. The 1D-^1^H-nuclear Overhauser effect spectroscopy (1D-NOESY) pulse sequence with presaturation during relaxation delay and mixing time and spoil gradient from the Bruker library was utilized (noesygppr1d). Acquisition parameters were set as follows: mixing time, 100 ms (d8); recovery delay, 2 s (d1); 90° pulse, 11.77 µs (p1); spectral width, 7,211.539 Hz (swh); spectral size, 32 k (td); number of scans, 256 (ns); and acquisition time, 2.27 s (aq).

Spectra were processed and analyzed using Chenomx 8.0 profiler software (Chenomx Inc., Edmonton, Canada). This software contains tools for automatic phase, baseline correction, reference calibration, and a library of metabolites for spectral profiling. Automatic tools were used, with manual corrections performed when required. The concentration of each identified metabolite was determined based on internal TSP concentration in the NMR tube (4.3 mM). Metabolic assignments and ^1^H spectral images are provided in Figures S1–S3 (see online supplementary material for a color version of these figures).

### Liver glycogen concentration

Frozen liver tissue samples were weighed and minced into small pieces. Each sample (200 mg of tissue) was homogenized in 1 mL of Assay Buffer (1X) from the Glycogen Assay Kit (Cayman Chemical, Michigan, USA, Item No. 700480) containing protease inhibitors: Nuclear Extraction Protease Inhibitor Cocktail (100X) (Cayman Chemical, Michigan, USA, Item No. 100009303) diluted 100-fold in the diluted Assay Buffer. The homogenate was centrifuged at 800 × *g* for 10 min at 4 °C, and the resulting supernatant was transferred to a new tube and stored on ice. Immediately after, glycogen concentrations were determined according to the manufacturer’s instructions for the Glycogen Assay Kit. Plates were read fluorometrically in Spark 10 m and software Sparkcontrol 2.1 (TECAN Austria GmbH, Grödig, Austria).

### Jejunal gene expression analysis

Frozen jejunal tissue samples were homogenized in Lysing Matrix D 2 mL tubes (MP Biomedicals, Irvine, California, USA) using a Precellys Evolution tissue homogenizer (Bertin Technologies, Montigny-le-Bretonneux, France) coupled with a Cryolys Evolution cooling system (Bertin Technologies, Montigny-le-Bretonneux, France), under a homogenization cycle of 6,500 rpm for 30 s at 4 °C. Total RNA was extracted with the Maxwell® RSC simplyRNA Tissue Kit (Promega Corporation, Madison, Wisconsin, USA) on a Maxwell® RSC instrument (Promega Corporation, Madison, Wisconsin, USA), following the manufacturer’s instructions. RNA quantity and purity were determined with a NanoDropND-1000 spectrophotometer (NanoDrop products, Wilmington, Delaware, USA), and RNA integrity was evaluated with an Agilent Bioanalyzer-2100 (Agilent Technologies, Santa Clara, California, USA). All samples had an RNA integrity number greater than 7. Approximately 1 µg of total RNA was reverse transcribed to double-stranded cDNA using random primers and the High-Capacity cDNA Reverse Transcription kit (Applied Biosystems, Foster City, California, USA) in a 20 µL reaction. Thermal cycling conditions were 25 °C for 10 min, 37 °C for 120 min, 85 °C for 5 min, followed by a 4 °C hold.

Gene expression was assessed by high-throughput real-time qPCR using a 96.96 Dynamic Array IFC for Gene Expression (Standard Biotools Inc., South San Francisco, California, USA) on a Biomark HD system (Standard Biotools Inc., South San Francisco, California, USA). A pooled cDNA sample, representative of all the experimental conditions, was used to evaluate qPCR efficiency for each gene following the relative standard curve method. Triplicates of two-fold (1/5, 1/10, 1/20, 1/40, 1/80, 1/160) and four-fold (1/320, 1/1280, 1/5120) serial dilutions were included. Samples were analyzed in duplicate at a 1/20 dilution for the quantification of 44 genes (40 target genes and 4 housekeeping genes) related to intestinal health, selected based on published literature (González-Solé et al. 2020; Villagómez-Estrada et al. 2022). Additionally, four genes not previously reported in those studies were analyzed: Myeloid Differentiation Primary Response 88 (***MyD88***) and Nuclear Factor Kappa-light-chain-enhancer of activated B cells (***NF-Kb***), both involved in immune and inflammatory responses; SH3 Domain Containing Ringer Finger 2 (***SH3RF2***), involved in protein regulation and degradation; and Proline-Rich AKT1 Substrate 1 (***AKT1S1***), a nutrient transport coding gene. Primer sequences and detailed information for all genes are presented in Table S1.

Data collection and quality control were conducted using the Fluidigm Real-Time PCR Analysis software (v4.8.1; Standard Biotools Inc., South San Francisco, California, USA). Gene expression analysis was then performed with DAG Expression software (v1.0.5.6; Ballester et al. 2013). The relative standard curve method was applied to account for gene-specific qPCR efficiency, which was obtained from a serial dilution of a representative cDNA pool, yielding quantity values. Gene expression was normalized using the geometric mean of four housekeeping genes (*ACTB*, *B2M*, *GAPDH*, and *TBP*), generating normalized quantity values. Gene expression data were analyzed by applying the 2^−ΔΔCt^ method for relative quantification and using the sample with the lowest expression as a calibrator.

### Ileal histomorphometry

Ileal tissue samples were dehydrated, embedded in paraffin wax, sectioned at 4 µm, and stained with hematoxylin and eosin. Morphological measurements were performed using a light microscope (BHS, Olympus, Barcelona, Spain) following the method described by Nofrarías et al. (2006). Villus height (**VH**), crypt depth (**CD**), and the VH:CD ratio were measured in a blinded manner on 10 well-oriented villi and crypts per sample by the same person.

### Statistical analysis

All calculations and statistical analyses were conducted using the open-source software R v.4.4.0 (Posit Team 2024). Residuals were tested for normality with the Shapiro-Wilk test using the *ols_test_normality* function from the olsrr package (v.0.6.0; Hebbali 2024) and visually evaluated with normal probability plots. Production performance data and piglet traits at birth were analyzed using linear mixed-effects models (*lmer* function, lme4 package, v.1.1-35.3; Bates et al. 2015). The models included piglet category (IUGR, R-LBW, NR-LBW, and N) as a fixed effect and sow as a random effect. Piglet vitality was analyzed using a cumulative link model (*clm* function, ordinal package, v.2023.12-4.1; Christensen 2023), with vitality scores (0–3) treated as an ordered categorical response. The model included piglet category as a fixed effect, and threshold parameters were estimated under a flexible structure. Vitality distributions for each piglet category were visualized using stacked proportional bar plots generated with the ggplot2 package (v.3.5.1; Wickham 2016).

Laboratory data (i.e., serum IgG, albumin, and IGF-I concentrations; serum metabolites; liver glycogen; and ileal histomorphology) were analyzed using the same modeling approach as production performance data and piglet traits at birth. Individual metabolites data were normalized using logarithmic transformation (log10) and mean centering. Although logarithmic transformations were performed to compare the metabolic concentrations between categories, original means are presented in Table S2. Therefore, results are expressed as least squares means ± SEM, except for metabolites, which are expressed as means ± SEM from transformed data. The experimental unit was the individual pig. Differences were considered significant at *P *< 0.05, whereas 0.05 ≤ *P *< 0.10 was considered a tendency. Metabolomics data were analyzed using the web-based platform MetaboAnalyst (https://www.metaboanalyst.ca). Differences among piglet categories were assessed using a PERMANOVA test. Partial least squares discriminant analysis (**PLS-DA**) was performed to explore differential metabolites among groups. The Variable Importance for Projection (**VIP**) score was used to determine the influence of every single component on the principal scores.

Relative gene expression quantification values were checked for normalization using a log10 transformation, and all statistical analyses were performed with R v.4.4.0 (Posit Team 2024) and Bioconductor (Gentleman et al. 2004). A one-way ANOVA with piglet category as a fixed effect was conducted, and the Benjamini–Hochberg false discovery rate (**FDR** *q*-value) was calculated to control for multiple testing (Benjamini and Hochberg 1995). Pairwise post hoc category comparisons between categories were carried out using Tukey’s honest significant difference test (Tukey 1949). Statistical differences were considered significant at *P*- and *q*-values below 0.05 for the ANOVA and Tukey’s analyses and for the FDR, respectively. A principal component analysis (**PCA**) was performed with samples as cases and gene log10-expressions as variables. The PCA function from the FactoMineR library (v.2.11; Lê et al. 2008) was used for dimensionality reduction and visualization of the first two principal components. The variables factor map was restricted to genes showing cos^2^-qualities greater than 0.45 and showing significant differences. Two samples from IUGR piglets were excluded from the analysis due to low gene expression and insufficient quantification quality.

## Results

### Growth performance

Growth performance from birth to weaning and CI are presented in Table 1. Body weight at birth differed among categories (*P *< 0.01), with IUGR piglets being the lightest and N piglets the heaviest. Differences in BW remained at d 1, 7, and weaning (*P *< 0.01). However, the numerical differences between IUGR and R-LBW piglets were no longer different at d 7 and weaning (*P *= 0.10 and *P *= 0.49, respectively). Average daily gain (**ADG**) was lower (*P *≤ 0.03) in IUGR and R-LBW piglets compared with NR-LBW and N piglets throughout all periods. Colostrum intake also differed among categories (*P *< 0.01), with IUGR piglets consuming the least on average and N piglets the most, while R-LBW and NR-LBW piglets had intermediate intake levels.

**Table 1:** Body weight (BW) and average daily gain (ADG) from birth to weaning, and colostrum intake for four categories of pigs classified by birth weight as intrauterine growth restricted (IUGR), restricted low birth weight (R-LBW), non-restricted low birth weight (NR-LBW), and normal birth weight (N).

	Category					
Item	IUGR	R-LBW	NR-LBW	N	SEM	*P*-value
**BW,^1^ kg**						
** Birth**	0.65^d^	0.88^c^	1.06^b^	1.44^a^	0.017	<0.01
** Day 1**	0.73^d^	0.92^c^	1.13^b^	1.54^a^	0.033	<0.01
** Day 7**	1.22^c^	1.47^c^	1.84^b^	2.49^a^	0.102	<0.01
** Weaning**	4.08^c^	4.69^c^	5.68^b^	7.27^a^	0.411	<0.01
**ADG,^2^ kg**						
** First week, d 0 to 7**	78.8^c^	87.2^c^	117^b^	158^a^	13.73	<0.01
** Day 7 to weaning**	146^c^	166^c^	200^b^	249^a^	17.46	<0.01
** Lactation, d 0 to weaning**	128^c^	142^c^	174^b^	220^a^	15.07	<0.01
**Colostrum intake,^3^ g/piglet**	208^d^	272^c^	354^b^	493^a^	17.45	<0.01

1 BW at birth (*n* = 107 IUGR, 216 R-LBW, 383 NR-LBW, 1,284 N); BW d 1 (*n* = 35 IUGR, 170 R-LBW, 318 NR-LBW, 980 N); BW d 7 (*n* = 19 IUGR, 136 R-LBW, 301 NR-LBW, 958 N); BW at weaning (*n* = 14 IUGR, 115 R-LBW, 286 NR-LBW, 929 N).

2 ADG first week (*n* = 19 IUGR, 134 R-LBW, 298 NR-LBW, 951 N); ADG d 7 to weaning (*n* = 14 IUGR, 114 R-LBW, 285 NR-LBW, 921 N); ADG lactation (*n* = 14 IUGR, 113 R-LBW, 284 NR-LBW, 922 N).

3 Colostrum intake (*n* = 35 IUGR, 168 R-LBW, 312 NR-LBW, 970 N).

^a,b,c,d ^Significant differences (*P*-value < 0.05).

In terms of survival, differences were observed among categories (*P *< 0.01) throughout lactation (results not shown). Mortality was highest in IUGR piglets (78.5%), followed by R-LBW piglets (33.1%), NR-LBW piglets (10.9%), and N piglets (5.88%).

### Piglet traits at birth

Piglets morphological (i.e., CRL, BMI, PI, RBW) and physiological (i.e., RT) characteristics at birth differed among the four categories (Table 2). Crown-to-rump length increased progressively from IUGR to N piglets (*P *< 0.01), with R-LBW and NR-LBW showing intermediate values, which differed from each other and from the other categories (*P *< 0.01). Body mass index followed a similar pattern, with IUGR piglets having the lowest values and N piglets the highest (*P *< 0.01). Ponderal index differed between IUGR and N piglets (*P *< 0.01), but no differences were observed between R-LBW and NR-LBW piglets (*P *= 1.00) or between R-LBW and N piglets (*P *= 0.11). Relative birth weight also differed among categories (*P *< 0.01). Rectal temperature recorded within 30 min of birth was lowest in IUGR piglets and highest in N piglets (*P *< 0.01), with R-LBW and NR-LBW piglets showing intermediate values.

**Table 2:** Birth traits of piglets across four categories based on birth weight: intrauterine growth restricted (IUGR), restricted low birth weight (R-LBW), non-restricted low birth weight (NR-LBW), and normal birth weight (N).

	Category					
Item	IUGR	R-LBW	NR-LBW	N	SEM	*P*-value
***n***	107	216	383	1,284		
**Physical characteristics**						
** Crown-to-rump length, cm**	19.1^d^	20.5^c^	21.9^b^	24.0^a^	0.123	<0.01
** Body mass index, kg/m^2^**	17.5^d^	20.7^c^	22.1^b^	24.7^a^	0.225	<0.01
** Ponderal index, kg/m^3^**	92.0^c^	101^ab^	101^b^	103^a^	1.27	<0.01
** Relative birth weight, %**	−49.8^d^	−30.1^c^	−14.3^b^	13.8^a^	1.36	<0.01
**Rectal temperature**						
** Rectal temperature at birth,^1^ °C**	35.0^d^	35.8^c^	36.3^b^	37.0^a^	0.169	<0.01

1 The rectal temperature of each piglet was measured within 30 min of birth.

^a,b,c,d^ Significant differences (*P*-value < 0.05).

**Figure 1:**
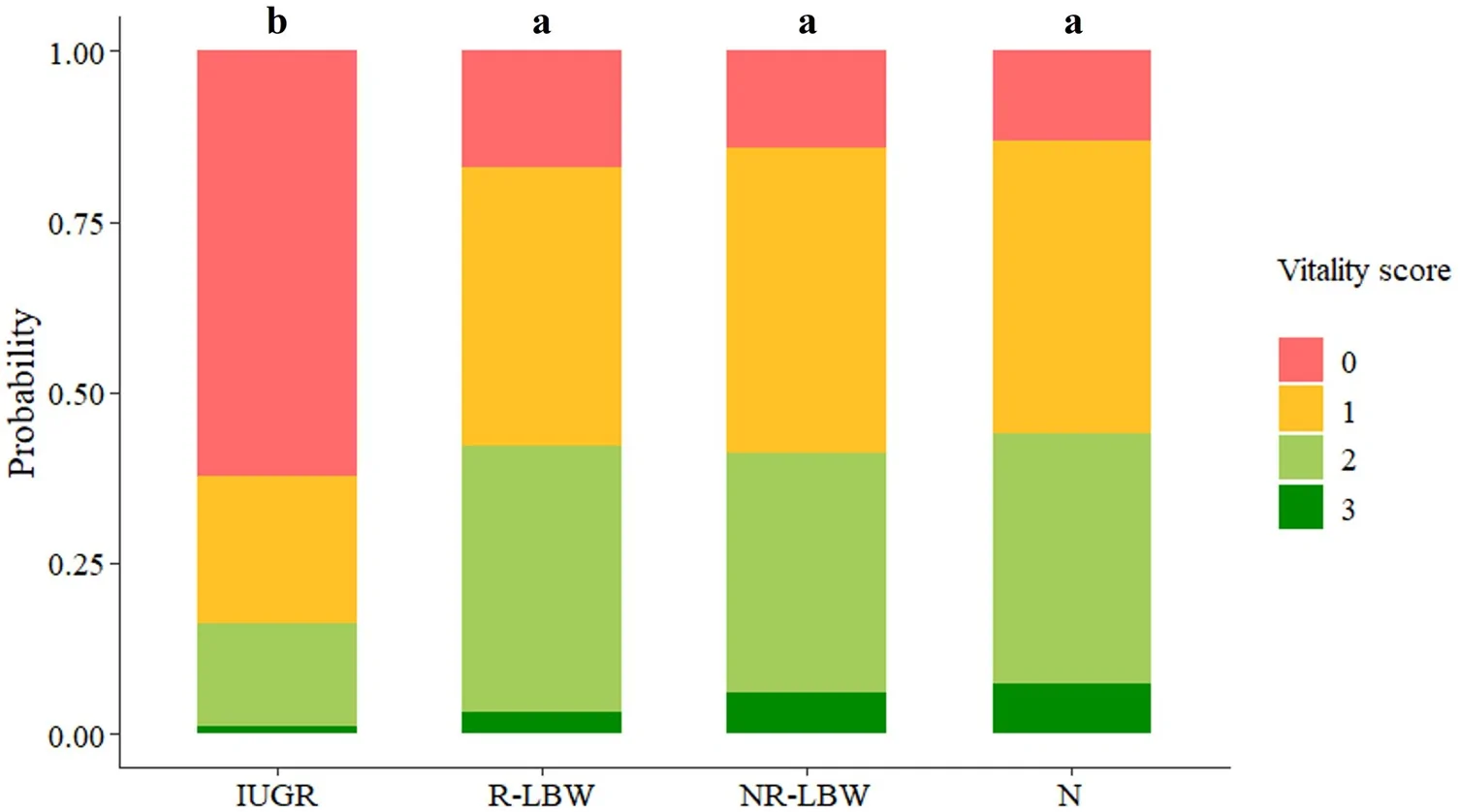
Probability distribution of vitality scores at birth for four categories of pigs classified by birth weight as intrauterine growth restricted (IUGR, *n* = 93), restricted low birth weight (R-LBW, *n* = 152), non-restricted low birth weight (NR-LBW, *n* = 294), and normal birth weight (N, *n* = 939). Significant differences among groups (*P *< 0.05) are indicated by different letters.

The probability distribution of vitality scores at birth is shown in Figure 1. Intrauterine growth restricted piglets exhibited the lowest vitality, with a greater proportion scoring 0 (62.4%) and 1 (21.5%) compared with the other categories (*P *< 0.01). In contrast, R-LBW, NR-LBW, and N piglets showed similar distributions (*P *≥ 0.66), with most piglets scoring 1 (40.8%, 44.6%, and 42.9%, respectively) or 2 (38.8%, 35.0%, and 36.4%, respectively). Only a small proportion of piglets in these three categories scored 0 (<18%) or 3 (<8%). The proportion of high-vitality piglets (score of 3) was low overall but was slightly greater in NR-LBW (6.1%) and N piglets (7.5%) compared with IUGR piglets (1.1%).

### Serum immunoglobulin G, albumin, and insulin-like growth factor I

Serum concentrations of IgG, albumin, and IGF-I at birth differed among piglet categories (Table 3). Immunoglobulin G concentrations were lowest in IUGR piglets and greatest in NR-LBW and N piglets (*P *< 0.01), with R-LBW piglets showing intermediate values. No differences were observed between NR-LBW and N piglets (*P *= 1.00). Albumin concentrations were lower in IUGR piglets compared with N piglets (*P *< 0.01), whereas R-LBW and NR-LBW piglets tended to differ from each other or from either of the extreme groups (0.06 ≤ *P *< 0.10). Insulin-like growth factor I concentrations were lowest in IUGR piglets and highest in N piglets (*P *< 0.01), with R-LBW and NR-LBW piglets showing intermediate values. Restricted-LBW piglets tended to differ from IUGR (*P *= 0.06) but did not differ from NR-LBW piglets (*P *= 0.27).

**Table 3:** Concentrations of immunoglobulin G (IgG), albumin, and insulin-like growth factor I (IGF-I) at birth for four categories of pigs classified by birth weight as intrauterine growth restricted (IUGR), restricted low birth weight (R-LBW), non-restricted low birth weight (NR-LBW), and normal birth weight (N).

	Category					
Item	IUGR	R-LBW	NR-LBW	N	SEM	*P*-value
***n***	12	12	12	12		
**Blood**						
** IgG, mg/mL**	1.281^c^	16.12^b^	27.94^a^	28.24^a^	3.180	<0.01
** Albumin, g/dL**	0.510^b^	0.618^ab^	0.636^ab^	0.731^a^	0.037	<0.01
** IGF-I, ng/mL**	23.97^c^	31.22^bc^	37.11^b^	54.02^a^	3.352	<0.01

^a,b,c^ Significant differences (*P*-value < 0.05).

### Overall metabolomic profile

**Figure 2:**
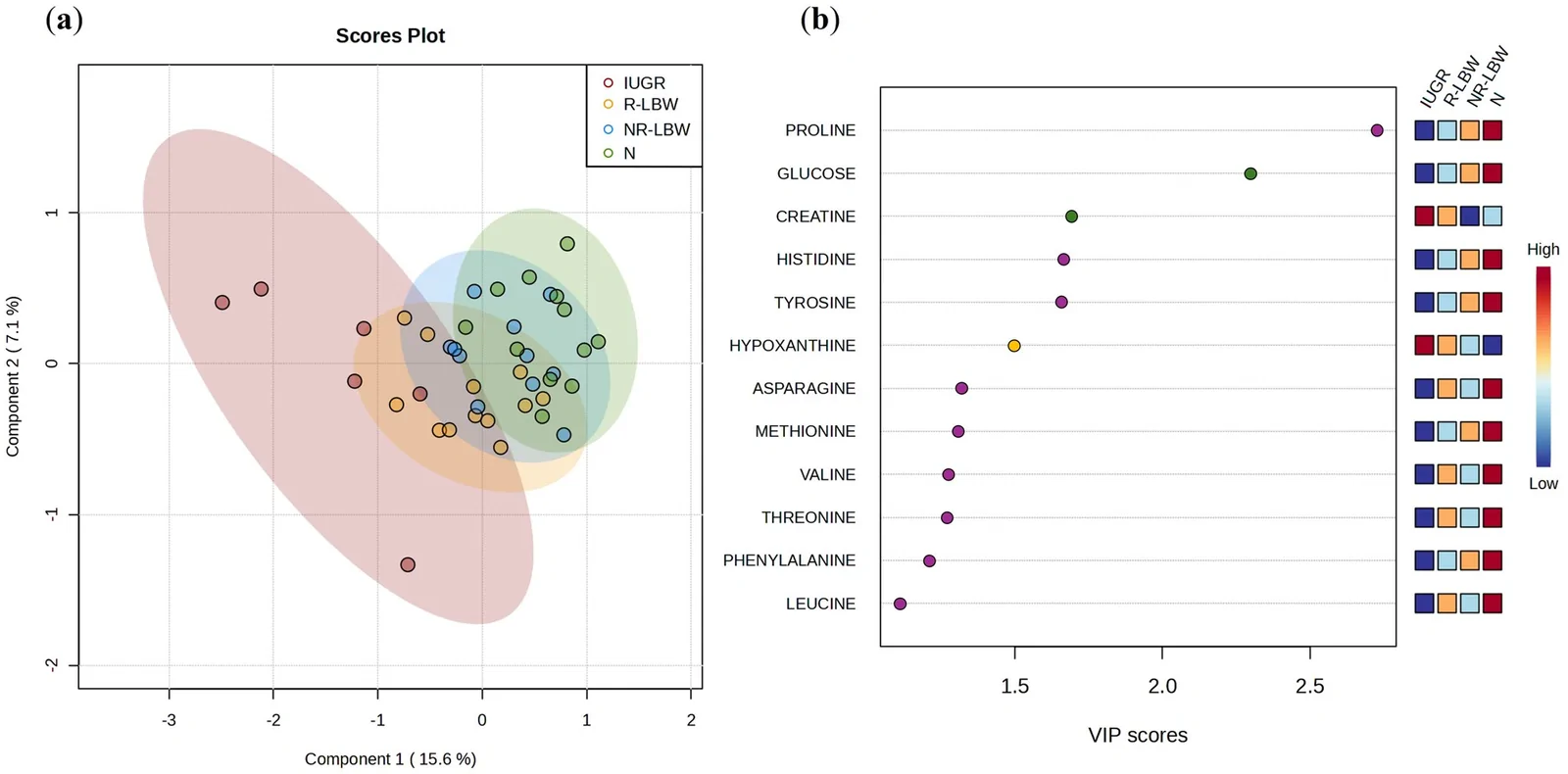
(a) PLS-DA 2D scores plot of serum metabolites from intrauterine growth restricted (IUGR, *n* = 6), restricted low birth weight (R-LBW, *n* = 12), non-restricted low birth weight (NR-LBW, *n* = 12), and normal birth weight (N, *n* = 12) piglets. Shaded areas in different colors indicate the 95% confidence intervals. (b) Metabolites with VIP scores >1. Metabolites are color-coded according to their biochemical category: amino acids (purple), energy metabolism-related metabolites (green), and purine metabolism-related metabolites (yellow).

The PLS-DA 2D score plot of serum metabolites revealed distinct clustering among the piglet categories (Figure 2a), with clear separation between IUGR piglets and the other categories. The PERMANOVA test confirmed a difference in the metabolic profile of IUGR piglets compared to the other categories (*P *≤ 0.01), whereas no differences were observed among the remaining three groups (*P *≥ 0.14) (results not shown). Metabolites with VIP scores > 1 are presented in Figure 2b, with amino acids (**AA**) contributing most to the cluster separation, followed by metabolites related to energy metabolism. Amino acids (i.e., Pro, His, Tyr, Asn, Met, Val, Thr, Phe, and Leu) were upregulated in N piglets compared to IUGR piglets (*P *≤ 0.03). Glucose concentrations also differed among categories (*P *< 0.01), being higher in N and NR-LBW piglets than in IUGR piglets. In contrast, hypoxanthine was upregulated in IUGR piglets compared to the other three categories (*P *< 0.01). Detailed metabolite concentrations for each category are provided in Table S2.

### Liver glycogen

**Figure 3:**
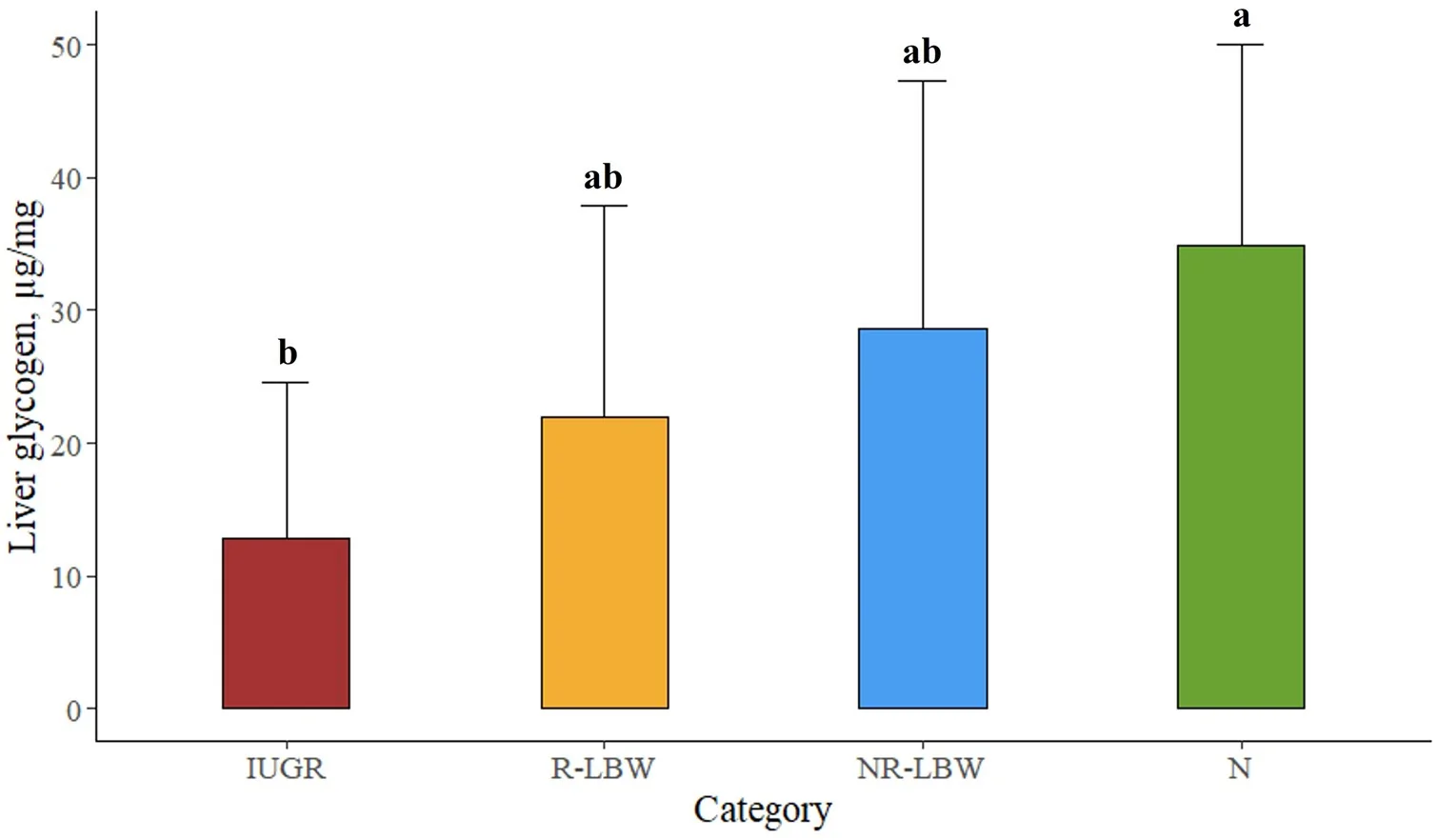
Liver glycogen concentration at birth for four categories of pigs classified by birth weight as intrauterine growth restricted (IUGR, *n* = 12), restricted low birth weight (R-LBW, *n* = 12), non-restricted low birth weight (NR-LBW, *n* = 12), and normal birth weight (N, *n* = 12). Significant differences (*P *< 0.05) are indicated by different letters.

The results of liver glycogen concentrations are presented in Figure 3. Differences were observed among the four categories (*P *= 0.01), with a linear increase from piglets with the lowest BtW to those with the highest. Intrauterine growth restricted piglets exhibited the lowest concentrations, whereas N piglets had the highest (*P *= 0.02). Restricted-LBW and NR-LBW piglets presented intermediate values that did not differ from each other or from the other categories (*P *≥ 0.13), due to the high variability within groups.

### Jejunal gene expression

The results of the jejunal gene expression analysis are presented in Table 4. Among antioxidant enzymes, *GPX2* was upregulated in N piglets compared with all other categories (*P *< 0.01), with IUGR piglets showing the lowest values and R-LBW and NR-LBW being intermediate and not different from each other (*P *= 0.85). In contrast, *SOD2m* expression was downregulated in NR-LBW piglets compared with IUGR piglets (*P *= 0.02). Genes associated with barrier function (*OCLN* and *ZO1*) were upregulated in IUGR piglets relative to all other categories (*P *< 0.01). A similar pattern was observed for *SH3RF2*, which was highest in IUGR piglets and lowest in NR-LBW and N piglets (*P *< 0.01). For digestive function, *ALPI* expression was upregulated in IUGR piglets compared with NR-LBW and N piglets (*P *≤ 0.03), while *DAO1* showed a comparable pattern. The *IGF1R* gene transcription was downregulated in NR-LBW and N piglets relative to IUGR piglets (*P *< 0.01), whereas R-LBW piglets displayed intermediate values that differed from the other three categories (*P *≤ 0.03). Immune-related genes also exhibited differential expressions: *GBP1* was downregulated in IUGR piglets compared with all other categories (*P *≤ 0.01), while *IFNGR1*, *NF-Kb*, *PPARGC1α*, *TGFβ1*, and *TLR4* were upregulated in IUGR piglets relative to N piglets (*P *≤ 0.04). Restricted-LBW and NR-LBW piglets showed intermediate expression levels that did not differ from each other. Several nutrient transport genes were upregulated in IUGR piglets, while N piglets consistently displayed the lowest expression (*P *≤ 0.03). Stress-related genes (*HSD11B1* and *CRHR1*) were upregulated in IUGR piglets compared with NR-LBW and N piglets (*P *≤ 0.02).

**Table 4:** Relative gene expression on jejunal mucosa at birth for four categories of pigs classified by birth weight as intrauterine growth restricted (IUGR, *n* = 10), restricted low birth weight (R-LBW, *n* = 12), non-restricted low birth weight (NR-LBW, *n* = 12), and normal birth weight (N, *n* = 12). Gene expression values are presented as ratios of cycle relative threshold value for each gene normalized to that of the reference sample. *P*-values come from the ANOVA test and FDR is the false discovery rate.

Function	Genes^1^	Category				SEM	*P*-value	*q*-value (FDR)
		IUGR	R-LBW	NR-LBW	N			
**Antioxidant enzyme**	*GPX2*	0.04^a,b,c^	0.39^a,b,c^	0.58^a,b,c^	1.81^a,b,c^	0.147	<0.01	<0.01
	*SOD2m*	1.31^a,b,c^	1.18^a,b,c^	0.75^a,b,c^	0.80^a,b,c^	0.054	0.01	0.02
**Barrier function**	*OCLN*	1.44^a,b,c^	1.14^a,b,c^	0.88^a,b,c^	0.79^a,b,c^	0.030	<0.01	<0.01
	*ZO1*	1.32^a,b,c^	0.88^a,b,c^	0.55^a,b,c^	0.51^a,b,c^	0.038	<0.01	<0.01
**Cell signaling regulation**	*SH3RF2*	1.66^a,b,c^	0.92^a,b,c^	0.66^a,b,c^	0.68^a,b,c^	0.032	<0.01	<0.01
**Digestive enzyme**	*ALPI*	1.50^a,b,c^	1.07^a,b,c^	0.94^a,b,c^	0.95^a,b,c^	0.041	0.02	0.04
	*DAO1*	1.28^a,b,c^	1.17^a,b,c^	0.83^a,b,c^	0.97^a,b,c^	0.040	0.02	0.04
**Digestive hormone**	*IGF1R*	1.57^a,b,c^	1.05^a,b,c^	0.75^a,b,c^	0.61^a,b,c^	0.035	<0.01	<0.01
**Immune response**	*GBP1*	0.46^a,b,c^	0.91^a,b,c^	0.89^a,b,c^	1.25^a,b,c^	0.069	<0.01	<0.01
	*IFNGR1*	1.13^a,b,c^	1.08^a,b,c^	0.76^a,b,c^	0.73^a,b,c^	0.043	<0.01	0.01
	*IL1β*	2.03^a,b,c^	0.42^a,b,c^	0.36^a,b,c^	0.28^a,b,c^	0.137	0.04	0.06
	*NF-Kb*	1.06^a,b,c^	0.88^a,b,c^	0.72^a,b,c^	0.67^a,b,c^	0.037	<0.01	0.01
	*PPARGC1α*	1.65^a,b,c^	1.16^a,b,c^	0.91^a,b,c^	0.94^a,b,c^	0.057	0.02	0.04
	*TGFβ1*	1.51^a,b,c^	1.20^a,b,c^	1.13^a,b,c^	1.13^a,b,c^	0.032	0.04	0.06
	*TLR4*	1.32^a,b,c^	1.14^a,b,c^	0.86^a,b,c^	0.87^a,b,c^	0.036	<0.01	0.01
**Nutrient transport**	*AKT1S1*	1.55^a,b,c^	1.07^a,b,c^	1.03^a,b,c^	0.87^a,b,c^	0.059	0.04	0.07
	*SLC15A1/PEPT1*	1.91^a,b,c^	1.40^a,b,c^	1.01^a,b,c^	0.99^a,b,c^	0.045	<0.01	<0.01
	*SLC16A1/MCT1*	1.16^a,b,c^	0.99^a,b,c^	0.78^a,b,c^	0.73^a,b,c^	0.048	0.02	0.04
	*SLC39A4/ZIP4*	1.87^a,b,c^	1.21^a,b,c^	1.04^a,b,c^	0.93^a,b,c^	0.057	0.03	0.05
	*SLC5A1/SGLT1*	1.69^a,b,c^	1.01^a,b,c^	0.54^a,b,c^	0.55^a,b,c^	0.088	<0.01	<0.01
	*SLC7A8*	2.49^a,b,c^	1.09^a,b,c^	0.78^a,b,c^	0.52^a,b,c^	0.077	<0.01	<0.01
**Stress enzyme**	*HSD11B1*	1.41^a,b,c^	1.16^a,b,c^	0.90^a,b,c^	0.89^a,b,c^	0.041	0.01	0.02
**Stress hormone**	*CRHR1*	3.20^a,b,c^	1.91^a,b,c^	1.65^a,b,c^	1.14^a,b,c^	0.067	<0.01	<0.01

1 Genes: *GPX2 *= glutathione peroxidase 2; *SOD2m* = superoxide dismutase 2; *OCLN* = occludin; *ZO1 *= zonula occludens 1; *SH3RF2 *= SH3 domain containing ringer finger 2; *ALPI* = intestinal alkaline phosphatase; *DAO1 *= D-amino acid oxidase; *IGF1R* = insulin like growth factor I receptor; *GBP1 *= guanylate binding protein 1; *IFNGR1 *= interferon gamma receptor 1; *IL1β* = interleukin 1 beta; *NF-Kb* = nuclear factor kappa-light-chain-enhancer of activated B cells; *PPARGC1α* = PPARG coactivator 1 alpha; *TGFβ1 *= transforming growth factor beta 1; *TLR4 *= toll like receptor 4; *AKT1S1 *= proline-rich AKT1 substrate 1; *SLC15A1/PEPT1 *= solute carrier family 15 member 1; *SLC16A1/MCT1 *= solute carrier family 16 member 1; *SLC39A4/ZIP4 *= solute carrier family 39 member 4; *SLC5A1/SGLT1 *= solute carrier family 5 member 1; *SLC7A8 *= solute carrier family 7 member 8; *HSD11B1 *= hydroxysteroid 11-beta dehydrogenase 1; *CRHR1 *= corticotropin releasing hormone receptor 1.

a,b,c Different letters in the same row indicate significant statistical mean differences in the four categories (Tukey’s test *P*-value <0.05).

**Figure 4:**
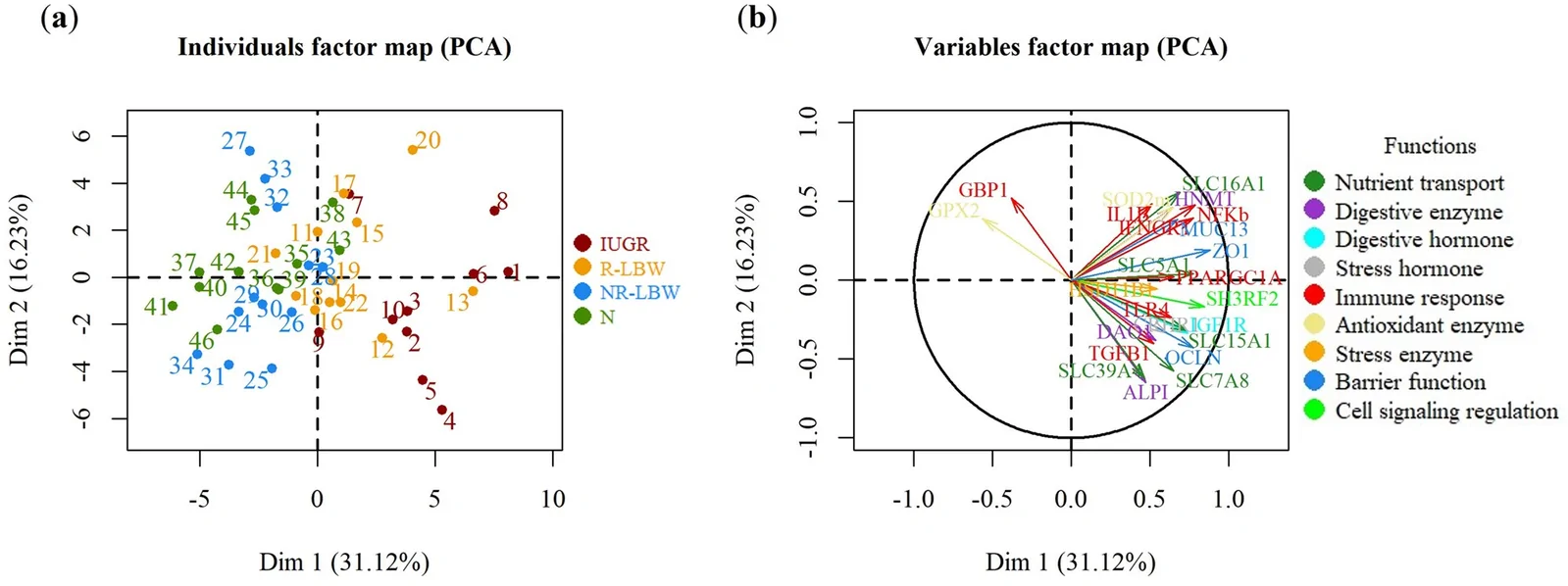
Principal component analysis (PCA). (a) Individuals factor map of jejunal samples, color-coded by category: IUGR = intrauterine growth restricted piglets (*n* = 10); R-LBW = restricted low birth weight piglets (*n* = 12); NR-LBW = non-restricted low birth weight piglets (*n* = 12); N = normal birth weight piglets (*n* = 12). (b) Variables factor map of gene expression in the jejunum. Only genes with *P *< 0.05 and represented by a long arrow are displayed. Each arrow color denotes a different functional group.

A PCA was performed to evaluate correlations in gene expression among samples from the four piglet categories. The individuals factor map (Figure 4a) displays sample identifiers colored by category, whereas the variables factor map (Figure 4b) illustrates the correlation structure of genes across samples. The 2D representation explained 47.35% of the total variance. Overall, the PCA revealed clear patterns in gene expression. All significant genes, except *GBP1* and *GPX2*, were well represented in the 2D space (long arrows) and were in the first and fourth quadrants. Samples from IUGR piglets (dark red) were also positioned in these quadrants, indicating medium to high expression levels. In contrast, *GBP1* and *GPX2* were positioned in the second quadrant, where samples from N and NR-LBW piglets (green and blue, respectively) were predominantly located. Samples from R-LBW piglets were dispersed across all quadrants.

### Ileal histomorphology

The ileal histomorphology results (Table 5) indicated differences in VH, CD, and the VH:CD ratio among piglets classified by BtW (*P *< 0.01). Normal piglets exhibited the greatest VH and CD values, whereas IUGR piglets showed the lowest values. Restricted-LBW and NR-LBW piglets had intermediate values. Specifically, NR-LBW piglets differed from IUGR piglets for both VH and CD (*P *≤ 0.01), while R-LBW piglets differed from IUGR and N piglets for VH (*P *≤ 0.01) and from N piglets for CD (*P *= 0.03). For the VH:CD ratio, IUGR piglets had the lowest value, N piglets had the highest, and both R-LBW and NR-LBW piglets exhibited intermediate values that did not differ from each other or from IUGR piglets.

**Table 5:** Ileal histomorphology at birth for four categories of pigs classified by birth weight as intrauterine growth restricted (IUGR), restricted low birth weight (R-LBW), non-restricted low birth weight (NR-LBW), and normal birth weight (N).

	Category					
Item	IUGR	R-LBW	NR-LBW	N	SEM	*P*-value
***n***	12	12	12	12		
**Ileal morphology**						
**VH, µm**	332^c^	426^b^	459^ab^	537^a^	23.3	<0.01
**CD, µm**	39.3^c^	43.4^bc^	46.1^ab^	47.7^a^	1.70	<0.01
**VH:CD ratio**	8.3^b^	9.6^b^	9.9^b^	11.7^a^	0.49	<0.01

^a,b,c^ Significant differences (*P*-value < 0.05).

## Discussion

This study investigated how internal characteristics differ among piglets classified into distinct BtW categories and evaluated whether these traits reflect different degrees of growth restriction, thereby influencing postnatal growth and survival potential. Consequently, it provides a comprehensive characterization of piglets across a range of BtW categories and highlights the biological and physiological challenges associated with IUGR. Extensive scientific literature has characterized piglets exhibiting severe IUGR at birth (Chaiyapatmaetee et al. 2025) and documented its consequences on preweaning performance (Van Ginneken et al. 2023), organ development and function (Bauer et al. 2003), and gastrointestinal health and maturation (Wang et al. 2005; Tang and Xiong 2022; Villagómez-Estrada et al. 2022), largely focusing on comparisons between severely IUGR and normal piglets. However, the present work emphasizes the importance of identifying piglets that do not display clear IUGR characteristics at birth but demonstrate poor subsequent growth, consistently remaining in the lowest quartile of the BW distribution and requiring additional time to reach market weight (Douglas et al. 2014; He et al. 2016; Montoro et al. 2020). This approach enables the distinction between constitutionally normal low BtW piglets, which are considered to have achieved their genetic growth potential (Ashworth 2013) and may have a greater capacity for postnatal recovery, and low BtW piglets exhibiting some degree of IUGR, which present developmental impairments that compromise their viability (Rutherford et al. 2013). Supporting this concept, Font (2020) demonstrated that pairs of half-siblings with the same low BtW can exhibit differences in BW by the end of the nursery phase, with these differences persisting throughout the fattening phase. This evidence suggests that piglets with comparable BtW may experience varying degrees of growth restriction, with important implications for growth performance and herd uniformity throughout the production cycle.

Differences in BtW among the four piglet categories were also reflected in distinct physical characteristics at birth (i.e., CRL, BMI, and RBW), consistent with previous findings from our group (Salgado-López et al. 2025), which reported strong correlations between BtW and these traits. Several studies have identified BtW as a key predictor of postnatal performance (Gondret et al. 2005; Rehfeldt et al. 2008; Fix et al. 2010) and survival (Van der Lende and De Jager 1991; Roehe and Kalm 2000), whereas others have suggested that indicators of body conformation may provide more accurate predictions of both survival and growth (Hales et al. 2013; Huting et al. 2018; Hansen et al. 2019). In agreement with these findings, survival and growth performance during lactation differed among categories. The high mortality observed in IUGR piglets (78.5%) compared to the other categories may be attributed to their low vitality scores, consistent with the results of Muns et al. (2013). Regarding growth performance, no differences in ADG during the first week or across the entire lactation period were observed between IUGR and R-LBW piglets, which reached similar BW at d 7 and at weaning, although these were lower than those of NR-LBW piglets. This underscores the importance of investigating additional indicators to better understand the biological challenges faced by low BtW piglets and reflects that changes in piglets’ BW categories are common during the lactation period (Douglas et al. 2013; Blavi et al. 2021).

Rectal temperature was measured immediately after birth to better reflect intrauterine conditions rather than the piglet’s body temperature after exposure to the external environment. Rectal temperature also differed among categories, with N piglets averaging 2 °C higher than IUGR piglets and about 1 °C higher than R-LBW and NR-LBW piglets. These findings align with those of Baxter et al. (2008, 2009), who reported higher birth temperatures in surviving compared with non-surviving piglets, but contrast with other studies in which RT was not associated with piglet survival or growth (Tuchscherer et al. 2000; Casellas et al. 2004; Panzardi et al. 2013).

Results regarding CI confirmed previous findings that piglet colostrum consumption increases with BtW (Devillers et al. 2007; Declerck et al. 2017; Hasan et al. 2019; Quesnel et al. 2023), showing a linear increase from IUGR to N piglets. Moreover, other piglet characteristics, such as vitality immediately after birth, have been identified as important factors influencing their ability to ingest colostrum (Peltoniemi et al. 2021; Oliviero 2023). This helps explain why IUGR piglets in this study consumed the lowest amounts of colostrum, on average, 285 g less than N piglets. Adequate colostrum ingestion is crucial for neonatal survival and development, as it provides immunoglobulins for immune protection and energy for thermoregulation and growth (Devillers et al. 2004). Colostrum is particularly rich in IgG (Le Dividich et al. 2005; Peltoniemi et al. 2021), which is essential for piglets to acquire passive immunity. Consistent with Oliviero (2023), who reported that piglets with higher BtW have increased IgG concentrations in the blood, the highest IgG levels in this study were observed in NR-LBW and N piglets, followed by R-LBW (with approximately half the concentration) and IUGR piglets, whose values were close to zero. This finding suggests that, while IUGR piglets are unable to achieve adequate colostrum intake, low BtW piglets exhibit variable initial suckling behaviors. Colostrum intake in this subset of piglets is critical for their future survival, as they have reduced energy reserves and a lower capacity for thermoregulation (Ward et al. 2020).

Quesnel et al. (2023) demonstrated positive associations of albumin and IGF-I with CI, partially explained by the fact that concentrations of these variables are positively correlated with BtW. In line with these findings, we observed higher albumin and IGF-I concentrations in N piglets compared with IUGR piglets. For albumin, R-LBW and NR-LBW piglets exhibited intermediate concentrations and tended to differ from each other or from either the N or IUGR categories, suggesting that the reduction in albumin was not restricted to IUGR piglets but varied according to the degree of growth impairment. Likewise, IGF-I concentrations were intermediate in R-LBW and NR-LBW piglets, with R-LBW piglets tending to have higher concentrations than IUGR piglets, although they did not differ from NR-LBW piglets. These findings suggest that low BtW piglets without clear signs of IUGR exhibit an intermediate metabolic phenotype between normal and IUGR piglets. Albumin plays a key role in transporting various proteins in the blood, and reduced levels have been associated with compromised growth, overall health, nutritional status, and liver function (Doornenbal et al. 1986; Wang et al. 2020; Rymut et al. 2021). Insulin-like growth factor I promotes mitogenic activity in target tissues and plays a critical role in the regulation of tissue and organ development (Morise et al. 2008). Concentrations of both albumin and IGF-I have been recognized as reliable indicators of fetal growth and maturity at birth (Herpin et al. 1993; Canario et al. 2007; Gondret et al. 2018), with low levels potentially reflecting impaired developmental maturity.

Another consequence of this reduced maturity at birth, often resulting from fetal malnutrition and inadequate CI (Sanz-Cortés et al. 2013), was the lower hepatic glycogen concentration observed in IUGR piglets compared with N piglets, consistent with the findings of Vanden Hole et al. (2019). In our study, IUGR piglets exhibited hepatic glycogen concentrations approximately 60% lower than those of N piglets. Such low glycogen levels at birth may compromise the piglet’s ability to mobilize their energy reserves, likely because these reserves are already critically low at birth. Consequently, impaired gluconeogenesis combined with poor glycogen stores results in low blood glucose concentrations, which have been associated with reduced viability and increased mortality (Panzardi et al. 2013). In agreement with these findings, we observed higher blood glucose concentrations in NR-LBW and N piglets compared with IUGR piglets, whereas R-LBW piglets showed intermediate values that did not differ from the other categories. Additionally, the lack of energy substrates in IUGR piglets appeared to numerically activate creatine metabolism, a by-product of phosphocreatine turnover. The non-significant elevated creatine levels observed in IUGR piglets may reflect a metabolic adaptation to compensate for insufficient energy availability (Sanz-Cortés et al. 2013), given that creatine is a key metabolite in energy metabolism through ATP production. In the present study, both glucose and creatine emerged as key metabolites associated with energy metabolism.

The main differences in the metabolic profile of IUGR piglets compared with the other categories were primarily due to a downregulation of AA. This finding aligns with Huang et al. (2019), who reported disturbances in AA metabolism in IUGR piglets 12 h after birth, which may contribute to the development of metabolic diseases later in life. Proline exhibited the highest VIP score, with N, NR-LBW, and R-LBW piglets showing higher concentrations than IUGR piglets. Proline plays a pivotal role in cell structure, antioxidative reactions, immune responses, energy metabolism, and protein synthesis (Wu et al. 2011). Its higher levels in piglets with greater BtW may be linked to CI, as Pro is one of the most abundant AA in sow colostrum (Wang et al. 2021). Phenylalanine and Tyr (synthesized from the essential AA Phe) were also reduced in IUGR piglets. Similarly, studies on severe IUGR cases reported decreased Phe levels (Paolini et al. 2001), attributed to impaired placental transport, likely resulting from placental tissue damage and the hypercatabolic state inherent to IUGR (Paolini et al. 2001; Lin et al. 2012). In agreement with Cetin et al. (1990) and Sanz-Cortés et al. (2013), the branched-chain AA Leu and Val were reduced in IUGR piglets. The literature identified chronic malnutrition and increased glucocorticoid exposure as potential mechanisms underlying the reduction in these AA concentrations (Hiroshige et al. 2001; Palova et al. 2007). Interestingly, the low concentrations of Leu and Tyr observed in IUGR piglets have been associated with reduced growth, altered metabolism, and impaired maturation of the nervous system (Ruggeri et al. 2025). Lower concentrations of Asn may also be linked to the reduced growth of IUGR piglets and their increased susceptibility to disease (Wu et al. 2006; Farmer and Edwards 2022). Although not observed within the first 24 h after birth, Ruggeri et al. (2025) reported lower Asn concentrations in IUGR compared with N piglets at the end of the starter period. Asparagine participates in energy and AA metabolism and protein synthesis (Wang et al. 2015) and supports immune function, benefiting the intestine and liver during inflammation (Wu et al. 2015; Chen et al. 2016). Other AA, such as His, Met, and Thr, which play crucial roles in protein synthesis and immune responses and possess antioxidative, anti-inflammatory, and immunomodulatory properties (Zhang et al. 2019a; Souza et al. 2023; Zheng et al. 2024) were also downregulated in IUGR piglets. Altogether, these AA are important for growth, protein synthesis, and neuromuscular development. Their reduced concentrations in IUGR piglets compared with the other categories likely reflect impaired placental transport of nutrients during gestation and decreased intestinal absorption. Additionally, alterations in liver and muscle metabolism may occur, as evidenced by reduced protein synthesis and changes in AA oxidation (Sanz-Cortés et al. 2013).

Conversely, hypoxanthine was upregulated in IUGR piglets compared with the other three categories. Hypoxanthine, a byproduct of purine metabolism, serves as a marker of hypoxia and oxidative stress. Elevated concentrations have been reported in hypoxic tissues of IUGR piglets, as blood flow tends to prioritize vital organs such as the brain and heart over peripheral tissues (Choi et al. 2025).

The integrity of intestinal morphology and function was also assessed at birth, as both are critical for intestinal health and nutrient utilization. Several studies have indicated that IUGR is associated with impaired intestinal development (Wang et al. 2005; Zhang et al. 2019b; Niu et al. 2021), linking the slow growth of IUGR piglets to intestinal injury (Tang and Xiong 2022). In the present study, IUGR and R-LBW piglets exhibited decreased VH and CD compared with N piglets, whereas the VH:CD ratio was higher in N piglets than in the other three categories. The results for VH and the VH:CD ratio are consistent with previous studies that also reported IUGR-related damage to the intestinal morphology of piglets at birth (D’Inca et al. 2010; Dong et al. 2014). In contrast, those studies observed either no differences or an increase in CD in IUGR piglets. Interestingly, Cui et al. (2022) and Tang and Xiong (2022) reported similar intestinal morphological differences by the end of the suckling period, suggesting that the impaired intestinal development and function observed at birth may have a lasting effect.

The maintenance of intestinal function is closely associated with normal intestinal morphology and structure, as well as with the integrity of the intestinal barrier and redox status (Chen et al. 2021a, 2021b). Consequently, impaired morphology can lead to altered intestinal function, as reflected by the disrupted gene expression patterns observed in IUGR piglets, which were similar to those of R-LBW piglets but differed from NR-LBW and N piglets. Newborns are particularly vulnerable to oxidative stress due to their immature antioxidant systems (Robles et al. 2001; Friel et al. 2004), with low BtW piglets being the most affected, as they exhibit reduced antioxidant enzyme activity compared with N piglets (Chen et al. 2021a, 2021b; Tang and Xiong 2022). Consistent with these findings, in the present study, N piglets showed the highest mRNA expression of the antioxidant enzyme *GPX2*, which helps eliminate reactive oxygen species and thus prevent oxidative stress (Circu and Aw 2010). However, no differences between IUGR and N piglets were observed for *SOD2m*, another gene involved in the detoxification of reactive oxygen species. Along with the oxidative stress observed in the small intestine of low BtW piglets, intestinal gene expression at birth showed an overexpression of stress-related (*HSD11B1* and *CRHR1*) and pro-inflammatory genes (*IL1β* and *NF-Kb*). These findings suggest that the first 24 h after birth represent a particularly stressful period for low BtW piglets, triggering an intestinal inflammatory response. Villagómez-Estrada et al. (2022) reported an overexpression of the stress-related gene *HSD11B1* in low BtW piglets. This gene encodes the enzyme that converts inactive cortisone into active cortisol, thereby regulating tissue glucocorticoid levels and playing a pivotal role in both metabolism and inflammatory response (Nixon et al. 2012). Although some studies have reported increased expression of pro-inflammatory pathways in IUGR piglets compared with N piglets (Tang et al. 2022; Villagómez-Estrada et al. 2022), their activation remains unclear, as other reports have described reduced levels of pro-inflammatory genes in IUGR neonates (Zhong et al. 2012; Dong et al. 2014). The simultaneous upregulation of stress-related and pro-inflammatory genes observed in our study suggests that low BtW piglets experience considerable physiological stress at birth. Interestingly, the *AKT1S1* gene, which encodes a subunit of mTORC1 (Wang et al. 2008), was upregulated in IUGR piglets compared with N piglets. This gene interacts with and inhibits mTORC1 activity (Yang et al. 2017), potentially disrupting mTORC1-dependent processes, thereby impairing protein synthesis, cell growth, and nutrient sensing and utilization in the jejunum (Wang et al. 2008). Similarly, the *SH3RF2* gene, which is involved in protein breakdown, was downregulated in NR-LBW and N piglets compared with the other categories. Remus et al. (2022) reported that this gene was downregulated in pigs with high protein deposition, suggesting that reduced expression favors protein synthesis while limiting protein breakdown.

Several studies have shown that oxidative stress is associated with intestinal barrier dysfunction and various gastrointestinal diseases (Navarro-Yepes et al. 2014; Zha et al. 2020; Chen et al. 2021a, 2021b). Wang et al. (2016) reported that neonates with IUGR exhibited increased tight-junction permeability and markedly reduced expression of occludin. In contrast, we observed an upregulation of tight-junction proteins (*OCLN* and *ZO1*) in IUGR piglets. These proteins form the intestinal mucosal barrier and are essential for regulating intestinal permeability (Chen et al. 2021a, 2021b). Moreover, in contrast with previous studies (Huang et al. 2020; Villagómez-Estrada et al. 2022), we found higher mRNA expression of digestive enzymes and hormones (*ALPI*, *DAO*, and *IGF1R*), as well as nutrient transport genes, in IUGR piglets. Although no previous studies have directly supported our findings, most research has examined newborn piglets before CI, whereas we assessed gene expression about 24 h after birth, when piglets had already consumed colostrum. In this context, Wang et al. (2018) demonstrated that intestinal proteins modulated by colostrum feeding play key roles in enhancing intestinal integrity, nutrient transport, energy metabolism, protein synthesis, and immune response. Nevertheless, colostrum only partially improved the compromised status of the jejunal mucosa in low BtW neonates, which still showed reduced expression levels compared with N counterparts. Further studies are needed to investigate compensatory mechanisms during early life, as evidence suggests that some low BtW piglets can catch up with their normal BtW counterparts by weaning (Cui et al. 2022).

## Conclusions

This study provides a comprehensive characterization of piglets across different birth weights, highlighting the biological and physiological challenges associated with intrauterine growth restriction. Piglets classified as intrauterine growth restricted exhibited the most severe impairments in postnatal growth, vitality, and survival, which were linked to reduced colostrum intake, limited energy reserves, altered metabolic profiles, and gastrointestinal dysfunction. Restricted and non-restricted low birth weight piglets also showed impaired growth and distinct morphological traits compared with normal birth weight piglets, although their physiological alterations were less pronounced and differences between these two categories were minimal. Our findings indicate that early-life classification based on birth weight provides valuable insights into the developmental status and growth potential of piglets. Future research should aim to refine predictive indicators of growth potential by considering not only intrinsic factors but also external influences, such as sow characteristics and competition within the litter. Early identification of low birth weight piglets at risk of growth retardation enables targeted management and nutritional interventions, potentially improving survival, growth performance, and overall production efficiency.

## Supplementary Material

skag258_Supplementary_Data

## Abbreviations

AA: amino acid
ADG: average daily gain
AKT1S1: proline-rich AKT1 substrate 1
BMI: body mass index
BtW: birth weight
BW: body weight
CD: crypt depth
CI: colostrum intake
CRL: crown-to-rump length
FDR: false discovery rate
IGF-I: insulin-like growth factor I
IgG: immunoglobulin G
IUGR: intrauterine growth restriction
MyD88: myeloid differentiation primary response 88
N: normal birth weight
NCC: number of completed circles around enclosure
NF-Kb: nuclear factor kappa-light-chain-enhancer of activated B cells
NMR: nuclear magnetic resonance
NR-LBW: non-restricted low birth weight
PBS: phosphate-buffered saline
PCA: principal component analysis
PI: ponderal index
PLS-DA: partial least squares discriminant analysis
RBW: relative birth weight
R-LBW: restricted low birth weight
RT: rectal temperature
SH3RF2: SH3 domain containing ringer finger 2
SID: standardized ileal digestible
U: udder stimulation
VH: villus height
VIP: variable importance for projection
