# Supplementary material for: Linking the degree of intrauterine growth restriction to alterations in metabolism, energy reserves, and gut health in newborn piglets
Source: J Anim Sci. 2026 Aug 17;104:skag258. doi: 10.1093/jas/skag258 (PMC13577159; doi:10.1093/jas/skag258)
Supplement: Supplementary file 1 [file skag258_supplementary_data.zip › Supplemental Material JAS_revision JAS-2025-10362.R3.docx]

| **Supplemental Table 1.** List of primers used for the gene expression analysis of 44 genes using the 96.96 Dynamic Array^TM^ IFC qPCR custom design. | | | | |
| --- | --- | --- | --- | --- |
| **Gene** | **Name** | **Forward Primer (5' → 3')** | **Reverse Primer (5' → 3')** | **Target/Housekeeping** |
| *ACTB* | Actin beta | CAAGGACCTCTACGCCAACAC | TGGAGGCGCGATGATCTT | Housekeeping |
| *AKT1S1* | Proline-rich AKT1 substrate 1 | CCACACAGCAGTATGCCAAGT | AGGAGGGCGGCCCAT | Target |
| *ALPI* | Intestinal alkaline phosphatase | ATGTCTTCTCTTTTGGTGGCTACA | GGAGGTATATGGCTTGAGATCCA | Target |
| *ANPEP* | Alanyl aminopeptidase membrane | AGGGCAACGTCAAAAAGGTG | GTCAAAGCATGGGAAGGATTTC | Target |
| *B2M* | Beta-2-microglobulin | TCACTCCTAACGCTGTGGATCA | CGGTTAGTGGTCTCGATCCC | Housekeeping |
| *CCK* | Cholecystokinin | CAGCAGGCTCGAAAAGCAC | AATCCATCCAGCCCATGTAGTC | Target |
| *CRHR1* | Corticotropin releasing hormone receptor | CAGGGCCCCATGATATTGG | CCGGAGTTTGGTCATGAGGAT | Target |
| *CXCL2* | Chemokine ligand | CATGGTGAAGAAAATCATCGAGAA | GCCAGTAAGTTTCCTCCATCTCTCT | Target |
| *DAO1* | D-amino-acid oxidase | GGAACCAACAGACCTTCAACTATCTC | TTCGGAATCCCAGGACCAT | Target |
| *FAXDC2* | Fatty acid hydroxylase domain containing | CCATGACTACCACCATCTCAAGTT | GCAGGATCGTGTGTCTCTCGTA | Target |
| *GAPDH* | Glyceraldehyde-3-phosphate dehydrogenase | TTCGTCAAGCTCATTTCCTGGTA | TCCTCGCGTGCTCTTGCT | Housekeeping |
| *GBP1* | Guanylate binding protein | AGAATCCATCACAGCAGACGAGTA | CGGATACAGAGTCGAGGCAGGTTAA | Target |
| *GPX2* | Glutathione peroxidase | CAACCAATTTGGACATCAGGAG | GGGTAAAGGTGGGCTGGAAT | Target |
| *HNMT* | Histamine N-methyltransferase | TGTTGAACCAAGTGCTGAACAAAT | ACTTTATGTTCTCGAGGTTTGATGTCTT | Target |
| *HSD11B1* | Hydroxysteroid 11-beta dehydrogenase | GGTCAGAAGAAACTCTCAAGAAGGTG | GCGAAGGTCATGTCCTCCAT | Target |
| *HSPA4/HSP70* | Heat shock protein 4 | TCAATTGCCTGCGATTAATGAA | GAATGCCCCATGTCTACAAAAAC | Target |
| *HSPB1/HSP27* | Heat shock protein 1 | CGAGGAGCTGACGGTCAAG | GCAGCGTGTATTTTCGAGTGAA | Target |
| *IFNGR1* | Interferon gamma receptor 1 | CATGTTACCCAAATCTTTGCTGTCT | CAGTATGCACGCTTGAAATTGTC | Target |
| *IGF1R* | Insulin-like growth factor | CCGACGCGGCAACAAC | TCAGGAAGGACAAGGAGACCAA | Target |
| *IL1β* | Interleukin 1 beta | GGTGACAACAATAATGACCTGTTATTTG | GCTCCCATTTCTCAGAGAACCA | Target |
| *IL10* | Interleukin 10 | TGAGGCTGCGGCGCT | GAGCTTGCTAAAGGCACTCTTCA | Target |
| *IL8* | Interleukin 8 | GGAAAAGTGGGTGCAGAAGGT | GAGAATGGGTTTTTGCTTGTTGT | Target |
| *MUC13* | Mucin 13 | CAGTGGAGTTGGCTGTGAAAAC | ATCAAGTTCTGTTCTTCCACATTCTTG | Target |
| *MUC2* | Mucin 2 | AAGGACGACACCATCTACCTCACT | GGCCAGCTCGGGAATAGAC | Target |
| *MyD88* | Myeloid differentiation primary response 88 | GATTACCTGCAAAGCAAGGAATG | TCTGATGGGCACCTGGAGAG | Target |
| *NF-Kb* | Nuclear factor kappa-light-chain-enhancer of activated B cells | CCCCACAGACGTTCATAGACAATTT | GAGGCTGGTTTTGTAATGTTGACA | Target |
| *OCLN* | Occludin | CAGGTGCACCCTCCAGATTG | CAGGCCTATAAGGAGGTGGACTT | Target |
| *PPARGC1α* | Peroxisome proliferative activated receptor gamma, coactivator 1 alpha | CTCTGGAACTGCAGGCCTAA | TGGAGAAGCCCTAAAAGGGTTAT | Target |
| *S100A9* | Calgranulin B | GAGCTGCCAAACTTTCTCAAGAA | AGTGTCCAGGTCTTCCAGGATGT | Target |
| *SH3RF2* | SH3 domain containing ring finger 2 | CCGAACGTCAGAATCCACATG | AGGATGACGTCTCCCTTATTGAAC | Target |
| *SI* | Sucrase-isomaltase | CGACCCCTTTTGCATGAGTT | AAGGCTGGACCCCATAGGAA | Target |
| *SLC13A1/NAS1* | Solute carrier family 13 (sodium/sulfate symporters) member 1 | GGTACCTCCACCAACTTGATCTTC | ATCCAAAGTTGATGCAGTGACAAT | Target |
| *SLC15A1/PEPT1* | Solute carrier family 15 (oligopeptide transporter) member 1 | GGTTATCCCTTGAGCATCTTCTTC | AGTGCTCTCATTCCATAGTAGGAAAA | Target |
| *SLC16A1/MCT1* | Solute carrier family 16 (monocarboxylic acid transporter) member 1 | CCTTGTTGGACCTCAGAGATTCTC | CCAGTATGTGTATTTATAGTCTCCGTATATGTC | Target |
| *SLC39A4/ZIP4* | Solute carrier family 39 (zinc transporter) member 4 | ATCTTTGGGCTCTTGCTCCTT | GCAGCCCCAGCACCTTAG | Target |
| *SLC5A1/SGLT1* | Solute carrier family 5 (sodium/glucose cotransporter) member 1 | GGCCATCTTTCTCTTACTGGCA | TCCCACTTCATGAAAAGCAAAC | Target |
| *SLC7A8* | Solute carrier family 7 (amino acid transporter light chain, L system) member 8 | TGTCGCTTATGTCACTGCAATGT | GACAGGGCGACGGAAATG | Target |
| *SOD2m* | Superoxide dismutase | GGGTTGGCTCGGTTTCAA | CATGCTCCCACACGTCGAT | Target |
| *TBP* | TATA-box binding protein | CAGAATGATCAAACCGAGAATTGT | CTGCTCTGACTTTAGCACCTGTTAA | Housekeeping |
| *TFF3* | Trefoil factor 3 | AGAACCTGCCCGTGACCAT | CACACTGGTTCGCCGACAG | Target |
| *TGFβ1* | Transforming growth factor beta 1 | GCGGCAGCTCTACATTGACTT | GACCTTGCTGTACTGAGTGTCTAGG | Target |
| *TLR4* | Toll-like receptor 4 | CATCCCCACATCAGTCAAGATACT | TCAATTGTCTGAATTTCACATCTGG | Target |
| *TNFα* | Tumor necrosis factor alpha | CACCACGCTCTTCTGCCTACT | GACGGGCTTATCTGAGGTTTGA | Target |
| *ZO1* | Zonula occludens 1 | GCTATGTCCAGAATCTCGGAAAA | TGCTTCTTTCAATGCTCCATACC | Target |

| **Supplemental Table 2.** Serum metabolite concentrations at birth in four categories of piglets classified by birth weight as intrauterine growth restricted (IUGR, n = 6), restricted low birth weight (R-LBW, n = 12), non-restricted low birth weight (NR-LBW, n = 12), and normal birth weight (N, n = 12). | | | | | | |
| --- | --- | --- | --- | --- | --- | --- |
|  | **Category** | | | |  |  |
| **Item** | **IUGR** | **R-LBW** | **NR-LBW** | **N** | **SEM** | ***P =*** |
| 2-Hydroxybutyrate | 0.012 | 0.015 | 0.014 | 0.012 | 0.003 | 0.79 |
| 2-Oxoglutarate | 0.038 | 0.039 | 0.041 | 0.038 | 0.005 | 0.94 |
| 3-Hydroxybutyrate | 0.007^b^ | 0.012^a^ | 0.014^a^ | 0.010^ab^ | 0.002 | < 0.01 |
| Alanine | 0.243^b^ | 0.414^a^ | 0.358^ab^ | 0.356^ab^ | 0.040 | 0.01 |
| Alloisoleucine | 0.011 | 0.020 | 0.024 | 0.020 | 0.004 | 0.04 |
| Asparagine | 0.019^b^ | 0.055^a^ | 0.062^a^ | 0.064^a^ | 0.010 | < 0.01 |
| Aspartate | 0.027 | 0.031 | 0.030 | 0.032 | 0.007 | 0.96 |
| Betaine | 0.266 | 0.347 | 0.384 | 0.261 | 0.051 | 0.03 |
| Choline | 0.022 | 0.022 | 0.022 | 0.021 | 0.003 | 0.95 |
| Citrate | 0.139^b^ | 0.155^ab^ | 0.185^ab^ | 0.195^a^ | 0.014 | 0.01 |
| Creatine | 0.084 | 0.059 | 0.053 | 0.054 | 0.022 | 0.48 |
| Creatinine | 0.048 | 0.052 | 0.048 | 0.052 | 0.004 | 0.55 |
| Formate | 0.037^b^ | 0.041^ab^ | 0.055^ab^ | 0.058^a^ | 0.006 | 0.01 |
| Glucose | 0.059^b^ | 1.43^ab^ | 2.57^a^ | 2.82^a^ | 0.567 | < 0.01 |
| Glutamate | 0.120 | 0.151 | 0.170 | 0.171 | 0.023 | 0.36 |
| Glutamine | 0.150^b^ | 0.276^a^ | 0.289^a^ | 0.336^a^ | 0.040 | < 0.01 |
| Glycerol | 2.24 | 2.13 | 1.74 | 1.98 | 0.307 | 0.36 |
| Glycine | 0.257^b^ | 0.423^a^ | 0.397^a^ | 0.406^a^ | 0.037 | < 0.01 |
| Histidine | 0.034^c^ | 0.075^b^ | 0.096^ab^ | 0.117^a^ | 0.014 | < 0.01 |
| Hypoxanthine | 0.062^a^ | 0.031^b^ | 0.021^b^ | 0.021^b^ | 0.010 | < 0.01 |
| Isobutyrate | 0.008^b^ | 0.018^a^ | 0.018^a^ | 0.018^a^ | 0.002 | < 0.01 |
| Isoleucine | 0.017 | 0.031 | 0.028 | 0.025 | 0.005 | 0.05 |
| Isopropanol | 0.008 | 0.012 | 0.012 | 0.009 | 0.001 | 0.02 |
| Lactate | 3.21 | 2.99 | 3.08 | 2.79 | 0.378 | 0.73 |
| Leucine | 0.027^b^ | 0.075^a^ | 0.068^a^ | 0.083^a^ | 0.011 | < 0.01 |
| Lysine | 0.072^b^ | 0.134^a^ | 0.133^a^ | 0.152^a^ | 0.020 | < 0.01 |
| Methanol | 0.065 | 0.069 | 0.066 | 0.075 | 0.012 | 0.87 |
| Methionine | 0.016^b^ | 0.028^ab^ | 0.031^ab^ | 0.039^a^ | 0.007 | 0.03 |
| Myoinositol | 3.29 | 1.88 | 2.94 | 1.86 | 1.41 | 0.44 |
| N,N-Dimethylglycine | 0.024 | 0.018 | 0.024 | 0.026 | 0.004 | 0.12 |
| Phenylalanine | 0.028^b^ | 0.061^a^ | 0.059^a^ | 0.067^a^ | 0.007 | < 0.01 |
| Proline | 0.082^b^ | 0.354^a^ | 0.415^a^ | 0.530^a^ | 0.073 | < 0.01 |
| Pyruvate | 0.168 | 0.211 | 0.199 | 0.178 | 0.027 | 0.53 |
| Serine | 0.106^b^ | 0.142^ab^ | 0.143^ab^ | 0.170^a^ | 0.016 | 0.01 |
| Succinate | 0.011 | 0.010 | 0.011 | 0.010 | 0.001 | 0.70 |
| Threonine | 0.040^b^ | 0.059^ab^ | 0.084^a^ | 0.090^a^ | 0.012 | < 0.01 |
| Tyrosine | 0.056^b^ | 0.171^a^ | 0.167^a^ | 0.186^a^ | 0.031 | < 0.01 |
| Urea | 0.582 | 0.797 | 0.637 | 0.765 | 0.097 | 0.23 |
| Valine | 0.066^b^ | 0.169^a^ | 0.169^a^ | 0.196^a^ | 0.025 | < 0.01 |

**Supplemental Figure legends**

**Supplemental Figure 1.** ^1^H-NMR spectrum (noesygppr1d) acquired at 600.13 MHz, 298 K, with a recovery delay of 2 s (d1), 256 scans (ns), and a mixing time of 100 ms (d8). Abbreviation: NMR = nuclear magnetic resonance.

**Supplemental Figure 2.** ^1^H-NMR spectrum (600.13 MHz). Expanded area from 0.8 to 4.3 ppm. Metabolite assignments were performed using the Chenomx 8.0 database. Abbreviation: NMR = nuclear magnetic resonance.

**Supplemental Figure 3.** ^1^H-NMR spectrum (600.13 MHz). Expanded area from 5.5 to 8.7 ppm. Metabolite assignments were performed using the Chenomx 8.0 database. Abbreviation: NMR = nuclear magnetic resonance.
